# P70. Development of clinically implementable imaging strategies for tracking T cell receptor-transgenic T cells

**DOI:** 10.1186/2051-1426-2-S2-P44

**Published:** 2014-03-12

**Authors:** S Mall, C D'Alessandria, M Aichler, A Walch, M Essler, M Schwaiger, C Peschel, A Krackhardt

**Affiliations:** 1 Klinikum rechts der Isar- Technische Universität München, III Medical Department, Munich, Germany; 2 Nuklearmedizinische Klinik und Poliklinik- Technische Universität München, Munich, Germany; 3 Helmholtz Zentrum München, Research Unit Analytical Pathology, Munich, Germany

## 

Transfer of T lymphocytes genetically modified with T-cell receptors (TCR) specific for tumour-associated antigens is a novel therapeutic approach for diverse malignant diseases. However, efficacy as well as safety of this therapy still needs to be improved. In order to understand T cell trafficking and functionality in vivo, the development of non-invasive and sensitive cell-tracking technologies would be of high value. Moreover, clinical translation of such technologies would further provide possibilities to improve this therapeutic approach in humans*.* We aim to establish a non-invasive, clinically translatable imaging approach for in vivo monitoring of adoptively transferred T cells engineered with selected TCRs. We have previously isolated several TCRs specifically recognising peptides derived from diverse tumor associated antigens and selected one TCR recognising lymphoma cells with defined HLA-DR restriction.

Peripheral blood mononuclear cells from healthy donors were retrovirally transduced with the selected TCR. To track transduced T cells, we used an anti-murine TCRβ monoclonal antibody (TCRmu), which binds to the murinzed region of the introduced TCR. This antibody was either radioiodinated with Iodine-124 or conjugated with a bifunctional chelator and labelled with Zirconium-89. Labelled antibodies were tested for stability and specific binding in vitro. A xenogenic mouse model was established using Nod/SCID mice injected intraperitoneally with lymphoma cells. After tumour inoculation, we transferred TCR-transduced human T cells and PET imaging was performed at different time points post injection of ^124^I-TCRmu or ^89^Zr-TCRmu. Specific in vivo binding was evaluated by co-injection of an excess of unlabelled antibody or isotype control antibody. In vivo uptake was confirmed by autoradiography and immunostaining for human CD3 on tumour frozen sections.

We established labelling of TCR transduced T cells using a specific antibody (TCRmu) marked with Iodine-124 or Zirconium-89. After the radio-labelling, affinity and specificity of the antibody was maintained while viability and functionality of T-cells remained unaffected. In vivo imaging of TCR-transduced T cells in the xenograft tumor model revealed strong uptake on the tumour area. Improved signal detection and reduced background was observed using ^89^Zr-anti-TCRmu. These results correspond to autoradiographic signals and detection of human T cells on the tumour border. Injection of an excess of unlabelled TCRmu showed depletion of human T cells in vivo, enabling a possible approach to control potentially autoreactive T cells in vivo.

In summary, we developed a non-invasive imaging model for tracking specifically human TCR-engineered lymphocytes in vivo. This model will be useful to monitor adoptive transfer of TCR transgenic T cells in vivo and therefore giving important information for further optimisations regarding efficacy and safety of immunotherapeutic approaches.

